# Differential roles of the ubiquitin proteasome system and autophagy in the clearance of soluble and aggregated TDP-43 species

**DOI:** 10.1242/jcs.140087

**Published:** 2014-03-15

**Authors:** Emma L. Scotter, Caroline Vance, Agnes L. Nishimura, Youn-Bok Lee, Han-Jou Chen, Hazel Urwin, Valentina Sardone, Jacqueline C. Mitchell, Boris Rogelj, David C. Rubinsztein, Christopher E. Shaw

**Affiliations:** 1 Institute of Psychiatry, King's College London, 1 Windsor Walk, Denmark Hill, London SE5 8AF, UK; 2 Department of Public Health, Neuroscience, Experimental and Forensic Medicine, University of Pavia, Via Ferrata 9, 27100 Pavia, Italy; 3 Jozef Stefan Institute, Department of Biotechnology, Jamova 39, 1000 Ljubljana, Slovenia; 4 Department of Medical Genetics, Cambridge Institute for Medical Research, University of Cambridge, Cambridge CB2 0XY, UK

**Keywords:** TDP-43, ALS, Autophagy, Proteasome, Aggrephagy, UPS

## Abstract

TAR DNA-binding protein (TDP-43, also known as TARDBP) is the major pathological protein in amyotrophic lateral sclerosis (ALS) and frontotemporal dementia (FTD). Large TDP-43 aggregates that are decorated with degradation adaptor proteins are seen in the cytoplasm of remaining neurons in ALS and FTD patients *post mortem*. TDP-43 accumulation and ALS-linked mutations within degradation pathways implicate failed TDP-43 clearance as a primary disease mechanism. Here, we report the differing roles of the ubiquitin proteasome system (UPS) and autophagy in the clearance of TDP-43. We have investigated the effects of inhibitors of the UPS and autophagy on the degradation, localisation and mobility of soluble and insoluble TDP-43. We find that soluble TDP-43 is degraded primarily by the UPS, whereas the clearance of aggregated TDP-43 requires autophagy. Cellular macroaggregates, which recapitulate many of the pathological features of the aggregates in patients, are reversible when both the UPS and autophagy are functional. Their clearance involves the autophagic removal of oligomeric TDP-43. We speculate that, in addition to an age-related decline in pathway activity, a second hit in either the UPS or the autophagy pathway drives the accumulation of TDP-43 in ALS and FTD. Therapies for clearing excess TDP-43 should therefore target a combination of these pathways.

## INTRODUCTION

Amyotrophic lateral sclerosis (ALS) and frontotemporal dementia (FTD) are neurodegenerative diseases that are characterised by the deposition of pathological protein aggregates composed of TAR DNA-binding protein 43 (TDP-43 or TARDBP) (Neumann et al., 2006). These ‘TDP-43 proteinopathies’ span a clinical spectrum, from predominantly upper- and lower-motor-neuron pathology in ALS to predominantly cortical-neuron pathology in FTD to a mixed presentation of both. TDP-43 is a DNA- and RNA-binding protein that regulates mRNA processing and trafficking, the stress granule response and microRNA biogenesis (Buratti et al., 2010; Colombrita et al., 2009; Gregory et al., 2004; Tollervey et al., 2011). A causative role for TDP-43 in disease was confirmed by the finding that mutations in TDP-43 cause ALS, accounting for ∼3% of familial and 0.5% of sporadic cases (Lagier-Tourenne and Cleveland, 2009). In the absence of genomic mutation, wild-type TDP-43 aggregates are found in the brain and spinal cord of 90% of ALS patients and in the brain of 60% of FTD patients (Neumann et al., 2006).

Evidence that the dysregulation of TDP-43 can cause disease comes from many animal models. A neurodegenerative ALS-like phenotype can be generated by either overexpression or knockdown of TDP-43 in flies, fish and mice (Kabashi et al., 2010; Li et al., 2010; Schmid et al., 2013; Wils et al., 2010; Wu et al., 2012). Indeed, various strands of evidence implicate a failure of TDP-43 clearance in human ALS and FTD. The aggregated TDP-43 that is present in ALS and FTD tissue is phosphorylated, ubiquitylated and labelled by ubiquilin-1 and -2 (Deng et al., 2011; Neumann et al., 2006) and sequestosome-1 (SQSTM1 or p62) (Arai et al., 2003), all of which target proteins for degradation. In addition, mutations in ubiquilin-2 (Deng et al., 2011), sequestosome-1 (Fecto et al., 2011) or another degradation pathway adaptor protein, valosin-containing protein (VCP) (Watts et al., 2004), can cause ALS and FTD characterised by TDP-43 aggregation. Cellular studies indicate that primary mutation of TDP-43 also confers resistance to its degradation (Ling et al., 2010).

Two of the major pathways for cellular protein degradation are the ubiquitin proteasome system (UPS) and macroautophagy, herein referred to as ‘autophagy’. Autophagy has been shown to degrade soluble and aggregated protein substrates that are too large to enter the UPS pore (Verhoef et al., 2002). Ubiquitylation can direct proteins towards either a UPS or an autophagic fate, therefore ubiquitin pathology in human ALS and FTD tissue does not distinguish which pathway is implicated in disease pathogenesis. The adaptor proteins sequestosome-1 and ubiquilin-2, which decorate TDP-43 aggregates, are also associated with both the UPS and autophagy (Lamark and Johansen, 2010).

Determining the relative importance of these two protein degradation pathways in maintaining TDP-43 proteostasis is challenging owing to the complex interplay between them, yet is crucial to understanding the mechanisms underpinning TDP-43 accumulation in disease. Although the role of TDP-43 macroaggregation remains controversial, the cytoplasmic accumulation of TDP-43 is definitely linked to toxicity, and therapeutic strategies designed to enhance TDP-43 clearance are of great appeal. We therefore sought to investigate how TDP-43 protein degradation is regulated and the potential for targeting degradative pathways as a treatment for ALS and FTD.

## RESULTS

### Establishing cellular models of TDP-43 proteinopathy

**Fig. 1.**
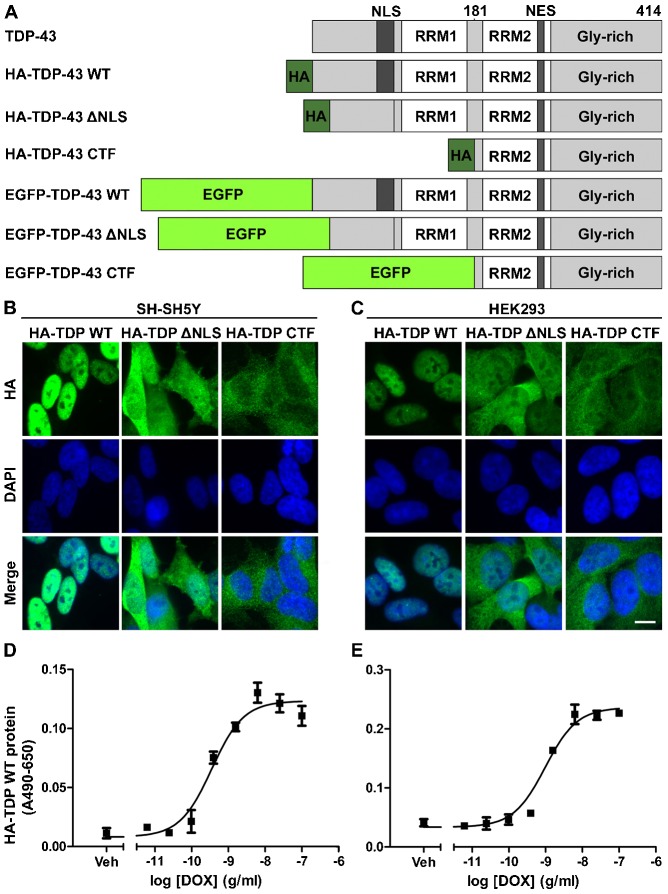
**Establishing cellular models of TDP-43 proteinopathy.** (A) A schematic of TDP-43 constructs used in this study. NES, nuclear export signal; RRM, RNA recognition motif. (B,C) Immunocytochemical analysis of TDP-43 expression and localisation in stable SH-SY5Y (B) and HEK293 (C) cell lines induced to express HA–TDP-43 constructs by a 48-h induction with DOX, showing nuclear localisation (WT), cytoplasmic localisation (CTF) or both (ΔNLS). Scale bar: 10 µm. (D,E) Cell-based ELISA validation of DOX-dose-dependent expression of HA–TDP WT in stable SH-SY5Y (D) and HEK293 (E) lines after a 48-h induction with DOX (EC_50_; HEK293, 0.97 ng/ml; SH-SY5Y, 0.35 ng/ml).

Stable cellular models of ALS and FTD based on doxycycline (DOX)-inducible expression of various TDP-43 constructs (Fig. 1A) were generated in human neuroblastoma (SH-SY5Y) cells and human embryonic kidney (HEK293) cells. In both cell types, and in agreement with previous reports, after induction with 1 µg/ml DOX, HA-tagged wild-type (WT) TDP-43 was predominantly partial nuclear, TDP-43 with a deleted nuclear localisation sequence (ΔNLS) was both nuclear and cytoplasmic, and a C-terminal fragment of TDP-43 (amino acids 181–414; CTF) was predominantly cytoplasmic (Fig. 1B,C). The partial nuclear localisation of ΔNLS TDP-43 is likely to be due to its dimerisation with endogenous TDP-43, leading to co-import. Stable SH-SY5Y and HEK293 lines expressing enhanced GFP (EGFP)-tagged TDP-43 WT, ΔNLS and CTF recapitulated these localisation profiles. The expression of TDP-43 in both SH-SY5Y and HEK293 lines was dependent on the dose of DOX (Fig. 1D,E, EC_50_; HEK293, 0.97 ng/ml; SH-SY5Y, 0.35 ng/ml).

### Degradation of TDP-43

Next, we sought to investigate the relative degradation rates of the TDP-43 constructs. Because of its ability to autoregulate (Ayala et al., 2011), quantification of endogenous TDP-43 protein degradation cannot be performed by steady-state analyses. We exploited the inducible nature of our cellular model to perform non-radioactive pulse–chase experiments, in order to assay the relative degradation of HA–TDP-43 protein after removal of the DOX inducer. In all experiments, HA–TDP-43 expression was induced for 24 h using 10 ng/ml DOX to achieve almost maximal expression.

**Fig. 2.**
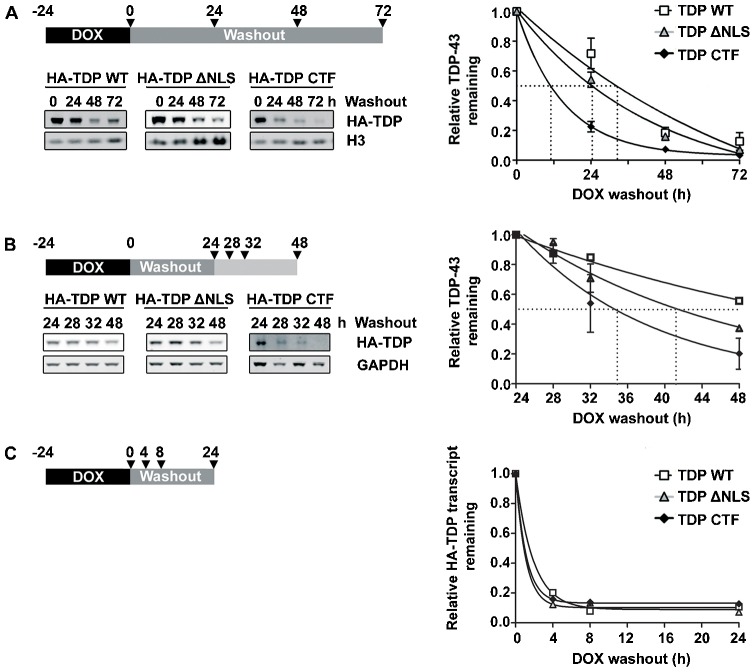
**Degradation of TDP-43.** (A) Degradation of HA–TDP-43 proteins in stable HEK293 cells as assessed by DOX pulse–chase experiments. TDP-43 CTF was degraded far more rapidly than TDP-43 ΔNLS or TDP-43 WT (*t*_1/2_; HA–TDP-43 WT, 32.5 h; HA–TDP-43 ΔNLS, 24.4 h; HA–TDP-43 CTF, 11 h). (B) Degradation of HA–TDP-43 proteins in stable SH-SY5Y cells over a shorter timecourse, and allowing a delay after washout for the clearance of mRNA transcripts. HA–TDP-43 protein-degradation rates closely reflected those seen in HEK293 cells (*t*_1/2_; HA–TDP-43 WT, 29.2 h; HA–TDP-43 ΔNLS, 16.6 h; HA–TDP-43 CTF, 10.2 h). (C) The degradation of HA–TDP-43 mRNA transcripts in stable SH-SY5Y cells as assessed by quantitative reverse transcriptase PCR. Transcripts for all three constructs decayed rapidly after DOX washout and had reached minima by 8 h (a representative experiment is shown, *n* = 2). (A–C) Arrowheads on schematics show the timepoints at which samples were taken.

In HEK293 lines assayed over 72 h (Fig. 2A), wild-type TDP-43 was degraded with a half-life of 32.5 h. The 25-kDa CTF of TDP-43, which is predominantly cytoplasmic, was turned over far more rapidly, with a half-life of 11 h. TDP-43 ΔNLS, which is both nuclear and cytoplasmic, showed an intermediate rate of degradation, with a half-life of 24.4 h. Previous estimates of the half-life of untagged wild-type TDP-43 have ranged from 4 h to ≥34 h (Ling et al., 2010; Pesiridis et al., 2011; Watanabe et al., 2013).

In order to verify that the assay was not simply measuring the dilution of HA–TDP-43 protein by cell division or the slow decay of DOX-induced transcripts, we performed a modified experiment over a shorter timecourse in SH-SY5Y lines (Fig. 2B), and used quantitative PCR to assess transcript levels after DOX washout (Fig. 2C). SH-SY5Y cells have a doubling time of ∼48 h, compared to ∼24 h for HEK293. In addition, protein levels were assayed over a 24-h period that began 24 h after DOX washout (see schematic Fig. 2B), to ensure that DOX-induced transcripts were not still present as a substrate for continued protein synthesis. Indeed, using real-time reverse transcriptase PCR, we verified that HA–TDP-43 transcripts rapidly decayed after DOX washout (Fig. 2C). Published half-lives for the endogenous TDP-43 transcript in various cell types range from 1.9 h to 10.3 h (Ayala et al., 2011; Schwanhäusser et al., 2011; Sharova et al., 2009). The three HA–TDP-43 proteins were turned over at very similar rates in SH-SY5Y cells compared to HEK293 cells, despite the differing cell division rates of these cell lines and the use of a shorter timecourse in experiments using SH-SY5Y cells (*t*_1/2_; HA–TDP-43 WT, 29.2 h; HA–TDP-43 ΔNLS, 16.6 h; HA–TDP-43 CTF, 10.2 h).

### Involvement of the UPS and autophagy in the degradation of TDP-43

**Fig. 3.**
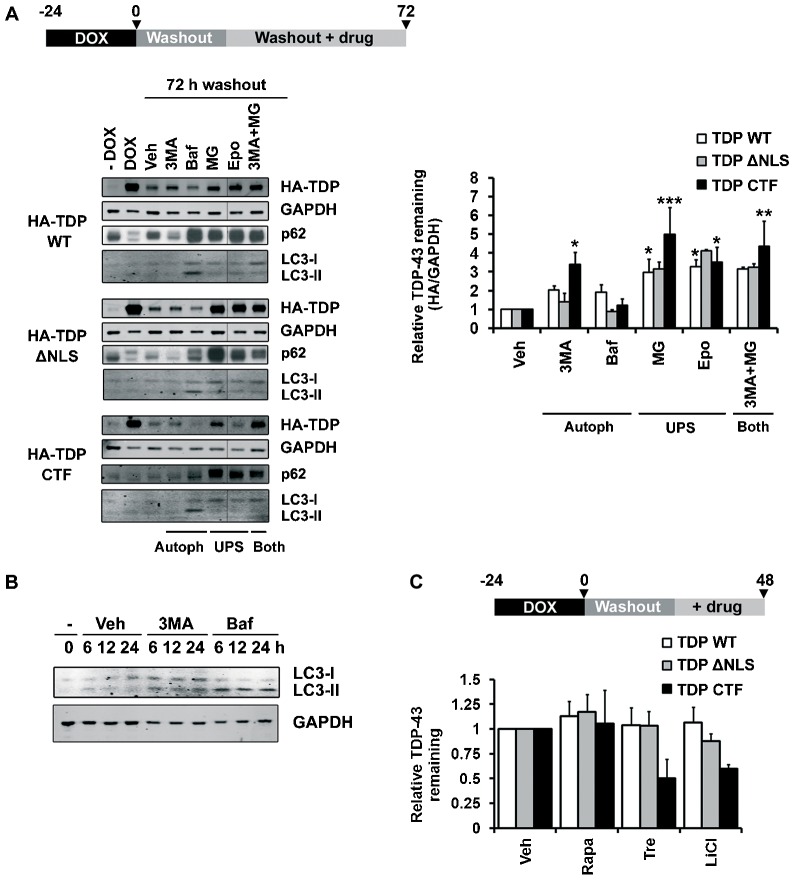
**Involvement of the UPS and autophagy in the degradation of TDP-43.** (A) The relative HA–TDP-43 remaining in stable HEK293 cells at 72 h post-DOX washout, with inhibitors of autophagy or the UPS included during the last 48 h. 3MA, 3-methyladenine; Baf, bafilomycin; Epo, epoxomicin; MG, MG132; Veh, vehicle. The −DOX control lane represents the amount of HA–TDP-43 present at the 72-h washout point without expression having been induced (‘leakage’). All lanes for each construct are from the same blot, spliced as shown. (B) Western blot of LC3 levels after short treatments with the autophagy inhibitors 3MA and bafilomycin showing LC3-I and II accumulation with 3MA and LC3-II accumulation with bafilomycin. (C) The relative HA–TDP-43 remaining in stable HEK293 cells subjected to DOX pulse–chase with activators of autophagy included during the last 24 h of the 48-h chase period. LiCl, lithium chloride; Rapa, rapamycin; Tre, trehalose; Veh, vehicle. The bars represent means±s.e.m. *P≤0.05, **P≤0.01, ***P≤0.001 between vehicle and inhibitor-treated cells (two-way ANOVA, Bonferroni post-test).

Having determined that TDP-43 degradation rates were consistent between cell lines and timecourses, we next analysed the pathway(s) by which TDP-43 was being degraded, using HEK293 lines. Inhibitors of the UPS or autophagy were included during the final 48 h of the DOX-washout period when protein degradation occurs (for schematic see Fig. 3A). Inhibitors were used in favour of genetic approaches in order to achieve temporal control and homogeneity across the cell population.

**Fig. 4.**
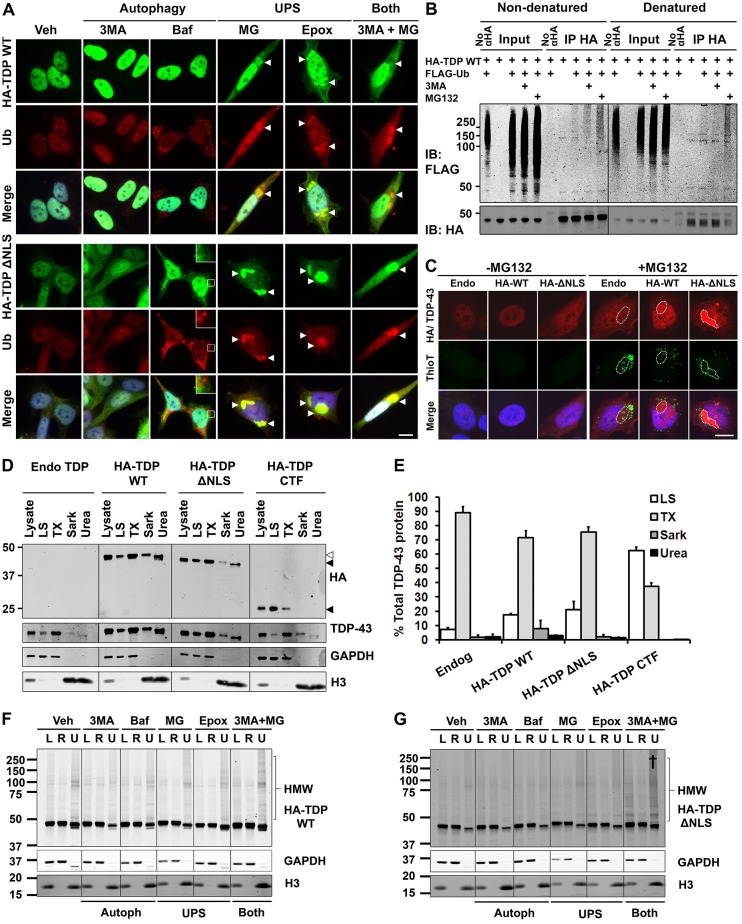
**Inhibiton of the UPS, but not autophagy, induces the formation of detergent-resistant cytoplasmic aggregates of TDP-43.** (A) Immunocytochemical analysis of stable SH-SY5Y lines induced to express HA–TDP-43 constructs by a 48-h induction with DOX in the presence of various inhibitors of the UPS or autophagy. When used alone, UPS inhibitors, but not autophagy inhibitors, induced the formation of ubiquitin (Ub)-positive TDP-43 macroaggregates (arrowheads). Scale bar: 10 µm. (B) Immunoprecipitation of HA–TDP-43 WT from HEK293 stable lines transfected with FLAG–Ub then induced with 1 µg/ml DOX alone or together with 3MA or MG132 for 24 h. Under non-denaturing conditions that preserve complexes (left) HA–TDP-43 WT co-immunoprecipitated with an increased amount of FLAG–Ub (smear >100 kDa) under treatment with 3MA and a greater amount again with MG132. Immunoprecipitation of denatured lysate (right) showed that HA–TDP-43 that was directly and covalently linked to FLAG–Ub only accumulated following MG132 treatment. IB, immunoblot. (C) TDP-43 aggregates are non-amyloid. Stable SH-SY5Y lines were induced to express HA–TDP-43 constructs by a 48-h induction with DOX in the absence or presence of MG132. In MG132-treated cells, thioflavin T staining detected amyloid aggregates that were independent of macroaggregates of HA–TDP-43 (dashed lines). MG132 treatment of parent SH-SY5Y cells induced macroaggregates (dashed line) with low levels of endogenous (Endo) TDP-43, likely owing to autoregulation of TDP-43 protein levels. Scale bar: 10 µm. (D,E) Four-step fractionation of stable SH-SY5Y lines induced to express HA–TDP-43 constructs by a 48-h induction with DOX. WT, upper white arrowhead; ΔNLS, middle black arrowhead; CTF, lower black arrowhead. The CTF was largely soluble in the low-salt (LS) fraction (TX, Triton X-100; Sark, sarkosyl). Note that the pellets from each step were resuspended in different volumes to ensure the detection of all fractions. Relative concentrations of the fractions are: Lys, 1; LS, 1; TX, 0.5; Sark, 3.33; urea 2.5. The bars represent means±s.e.m. (F,G) Solubility fractionation of the cells treated as in A, showing the lysate (L), RIPA-soluble (R) and urea-soluble (U) fractions of HA–TDP-43 WT (F) and ΔNLS (G). Urea fractions are concentrated 10-fold. Combined treatment with UPS and autophagy inhibitors (†) increased the amount of insoluble high-molecular-weight (HMW) TDP-43, particularly for TDP-43 ΔNLS (G). 3MA, 3-methyladenine; Baf, bafilomycin; Epox, epoxomicin; MG, MG132; Veh, vehicle.

These assays examined the total complement of TDP-43, of which ∼90% was detergent-soluble (Fig. 4D,E). The degradation of all three TDP-43 species was inhibited to varying degrees by the panel of inhibitors tested (Fig. 3A). The autophagy inhibitor 3-methyladenine (3MA) significantly inhibited the degradation of CTF TDP-43 (*P*≤0.05) and, to a lesser extent, the degradation of wild-type and ΔNLS TDP-43. 3MA showed minimal effect on the autophagy reporters p62 and LC3 (microtubule-associated protein 1 light chain 3 β) at the timepoint chosen for assay of TDP-43 levels (48 h 3MA); however, shorter timecourses of 3MA treatment revealed autophagy inhibition had occurred (Fig. 3B). The autophagosome–lysosome fusion inhibitor bafilomycin (Baf) only minimally inhibited the degradation of each TDP-43 species, despite effectively inhibiting autophagy as shown by the accumulation of LC3-II, the modified form of LC3 that associates with autophagosome membranes (Kabeya et al., 2000). In contrast to the moderate effects of autophagy inhibitors, the UPS inhibitor MG132 significantly inhibited the degradation of all three TDP-43 constructs (Fig. 3A, WT, *P*≤0.05; ΔNLS, *P*≤0.05; CTF, *P*≤0.001). The related UPS inhibitor epoxomicin also caused significant accumulation of wild-type, ΔNLS and CTF TDP-43 (all *P*≤0.05). The inhibition of both degradation pathways (by combined treatment with 3MA and MG132) did not inhibit the degradation of any of the protein constructs any more than MG132 alone. Activators of the autophagy pathway validated these findings because the addition of trehalose and LiCl during DOX washout substantially accelerated the degradation of CTF TDP-43 (Fig. 3C).

### UPS, but not autophagy, inhibition induces cytoplasmic TDP-43 macroaggregates

We sought to assess the phenotypic consequences of TDP-43 accumulation under either autophagic or UPS inhibition. Parallel biochemical and immunocytochemical studies were carried out in SH-SY5Y cells, as their large cytoplasm was more amenable to imaging than HEK293 cells.

To investigate the localisation and accumulation of TDP-43, its expression was induced with 1 µg/ml DOX in stable SH-SY5Y cells in the presence of inhibitors of the UPS or autophagy for 48 h. For both wild type and ΔNLS, inhibition of the UPS, but not autophagy, caused TDP-43 accumulation, resulting in the formation of macroaggregates (Fig. 4A). The UPS inhibitor MG132 induced the formation of large cytoplasmic aggregates in ∼20% of cells [WT, 17.4%±1.3 (224 cells counted); ΔNLS, 25.6%±3.4 (368 cells counted) (±s.e.m.)]. Epoxomicin also induced TDP-43 macroaggregates. By contrast, neither of the autophagy inhibitors 3MA or bafilomycin induced the formation of ubiquitylated TDP-43 macroaggregates. Interestingly, the co-application of MG132 and 3MA generated aggregates more robustly in cells expressing either HA–TDP-43 WT or HA–TDP-43 ΔNLS than did MG132 alone.

As shown in Fig. 1B,C, the HA–TDP-43 CTF was expressed at extremely low levels in both SH-SY5Y and HEK293 cells and was barely detectable by immunocytochemistry, likely because of its rapid degradation (as shown in Fig. 2A,B). Therefore, the localisation of the HA–TDP-43 CTF was not investigated further. We have found that an equivalent EGFP-tagged TDP-43 CTF forms aggregates under UPS inhibition but not under autophagy inhibition, similar to wild-type or ΔNLS TDP-43. However, the physiological relevance of the EGFP-tagged fragment is unclear, given that we and others (Li et al., 2011) find its steady state level to far exceed that of CTFs with small tags.

To examine more sensitively the conditions under which ubiquitylated TDP-43 accumulates, we performed co-immunoprecipitation after treatment with 3MA or MG132, under native or denaturing conditions (Fig. 4B). In HEK293 HA–TDP-43 WT stable lines transfected with FLAG-tagged ubiquitin (FLAG–Ub) and then induced with 1 µg/ml DOX alone, immunoprecipitation with anti-HA antibody yielded a small amount of FLAG–Ub, which ran as high-molecular-weight smears (>100 kDa). Induction with DOX plus 3MA for 24 h resulted in a small increase in the co-immunoprecipitation of FLAG–Ub by HA–TDP-43 WT, whereas treatment with MG132 for 24 h gave a more striking increase. This finding, which arose under non-denaturing immunoprecipitation conditions that preserve protein complexes, could reflect the formation of complexes containing HA–TDP-43 and ubiquitylated molecules, rather than direct ubiquitylation of HA–TDP-43. Indeed, using denaturing conditions (in which the lysate was boiled with 0.5% SDS to denature non-covalent interactions) a direct FLAG–Ub interaction with HA–TDP-43 was observed following MG132 treatment but not under basal or 3MA-treated conditions. These findings indicate that ubiquitylated TDP-43 accumulates predominantly when the UPS is blocked.

TDP-43 aggregates in human ALS and FTD were initially characterised as non-amyloid (Cairns et al., 2007); however, recent studies have found TDP-43 aggregates to be amyloid in at least a subset of cases (Bigio et al., 2013; Robinson et al., 2013). We used thioflavin T staining to determine whether the TDP-43 aggregates induced by UPS inhibition were amyloid. UPS inhibition induced the formation of multiple small amyloid aggregates, which were sometimes studded within TDP-43-positive macroaggregates; however, the TDP-43 macroaggregates themselves were negative for thioflavin T (Fig. 4C). These findings suggest that the macroaggregates are amorphous and non-amyloid.

We next investigated the relationship between macroaggregates detected by immunocytochemistry and detergent-insoluble species on western blot. Insoluble high-molecular-weight TDP-43 and insoluble fragmented TDP-43 have been described by Neumann and colleagues as hallmark features of ALS and FTD (Neumann et al., 2006). The sequential biochemical fractionation of TDP-43 proteins (generated by a 48-h induction of stable SH-SY5Y cells with 1 µg/ml DOX) demonstrated that, under basal conditions, HA-tagged wild-type or ΔNLS TDP-43 showed a similar biochemical profile to endogenous TDP-43, with a small proportion (7–21%) soluble in the low-salt fraction, the majority of the protein in the Triton X-100-soluble fraction (72–89%) and a minority of the protein soluble only in sarkosyl or urea (4–11%) (Fig. 4D,E). The HA-tagged CTF TDP-43 that was used in this study was almost completely soluble in low-salt and Triton X-100 fractions (>99%). Given its low expression levels, lack of aggregate formation and high basal solubility, the solubility of HA–TDP-43 CTF was not investigated further.

Using a simplified solubility protocol, previously validated to isolate insoluble aggregated TDP-43 (Winton et al., 2008), we analysed the effect of the inhibition of the UPS or autophagy on the solubility of wild-type and ΔNLS TDP-43 (Fig. 4F,G). As for the immunocytochemical experiments, TDP-43 expression in stable SH-SY5Y cells was induced with 1 µg/ml DOX in the presence of inhibitors of autophagy or the UPS for 48 h. The effect of UPS inhibitors on total TDP-43 levels (lysate) was more subtle than that shown in Fig. 3A, a result that is likely due to the differing experimental paradigm – the application of inhibitors during washout in Fig. 3A versus the application of inhibitors with continued overexpression in Fig. 4F,G. Owing to the low proportion of cells forming aggregates, we could not detect an increase in TDP-43 in the insoluble fraction under conditions which yielded immuno-detectable macroaggregates. However, combined UPS and autophagy inhibition (by treatment with 3MA and MG132) was shown to increase the level of insoluble high-molecular-weight (oligomeric) TDP-43, indicating that these species might be regulated by autophagy. Also of note, even under experimental conditions where TDP-43 appears diffuse by immunocytochemistry (Veh, 3MA, Baf) a significant amount of monomeric TDP-43 was insoluble in RIPA buffer.

### TDP-43 aggregates induced by UPS inhibition or dual inhibition of the UPS and autophagy resemble those seen in human disease

We next characterised TDP-43 macroaggregates in terms of their labelling by molecules that target them for degradation. Given that dual inhibition of the UPS and autophagy increased the levels of insoluble TDP-43 and enhanced macroaggregation more than UPS inhibition alone, we compared the labelling of aggregates induced by dual inhibition with the labelling of those induced by UPS inhibition alone.

**Fig. 5.**
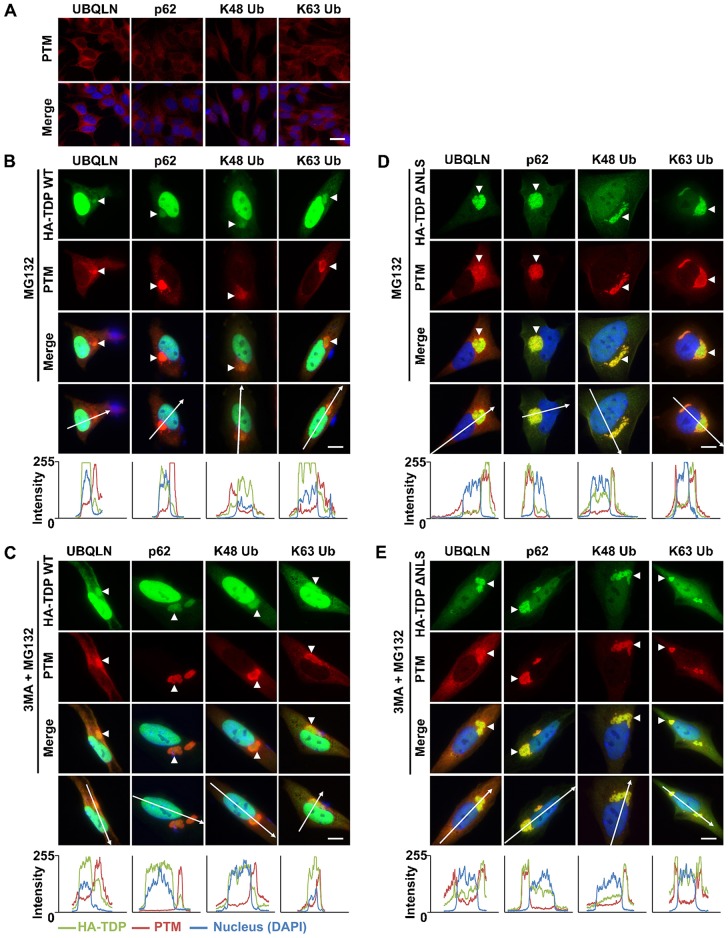
**TDP-43 aggregates induced by UPS inhibition or dual inhibition of the UPS and autophagy resemble those seen in human disease.** (A) Immunocytochemical analysis of untreated SH-SY5Y cells showing the diffuse localisation of the degradation pathway proteins ubiquilin-1 or -2 (UBQLN) and p62, and K48- and K63-linked forms of ubiquitin (Ub). Scale bar: 25 µm. (B–E) Immunocytochemical analysis of stable SH-SY5Y lines induced to express HA–TDP-43 by a 48-h induction with DOX in the presence of UPS inhibition or combined inhibition of the UPS and autophagy. (B) HA–TDP-43 WT formed cytoplasmic macroaggregates (arrowheads) following treatment with DOX plus the proteasome inhibitor MG132 in stable SH-SY5Y cells. These aggregates were positive for post-translational modifications (PTMs) including K48- and K63-linked ubiquitin, ubiquilin-1 and -2 and p62. (C) Combined treatment with DOX, MG132 and the autophagy inhibitor 3MA enhanced the recruitment of HA–TDP-43 WT to macroaggregates, which were positive for the same markers as with MG132 treatment alone. (D) HA–TDP-43 ΔNLS also formed cytoplasmic aggregates following a 48-h treatment with DOX plus MG132 in stable SH-SY5Y cells. As was seen for HA–TDP-43 WT, these aggregates were positive for K48- and K63-linked ubiquitin, ubiquilin-1 and -2 and p62. (E) Combined treatment with DOX plus MG132 and 3MA did not markedly alter the recruitment of HA–TDP-43 ΔNLS to macroaggregates. Arrows on the bottom row of images in B–E represent the section taken for the intensity profiles shown below the images. Scale bars: 10 µm.

We first used untreated SH-SY5Y cells to characterise the basal pattern of staining of the degradation adaptor proteins ubiquilin-1 and -2 (UBQLN) and p62 (two of which, UBQLN2 and p62, are seen in aggregates in human ALS and FTD), and polyubiquitin chains with different internal lysine linkages (K48 and K63) (Fig. 5A). These adaptors were localised diffusely in untreated cells but colocalised with aggregates of wild-type (Fig. 5B,C) or ΔNLS TDP-43 (Fig. 5D,E) that had been induced with 1 µg/ml DOX plus either MG132 with 3MA or MG132 alone. Although 3MA enhanced the recruitment of HA–TDP-43 WT to perinuclear aggregates induced by MG132 treatment (Fig. 5, intensity plots), it did not alter the labelling profile of the aggregates.

### TDP-43 macroaggregates are cleared upon the restoration of UPS function

**Fig. 6.**
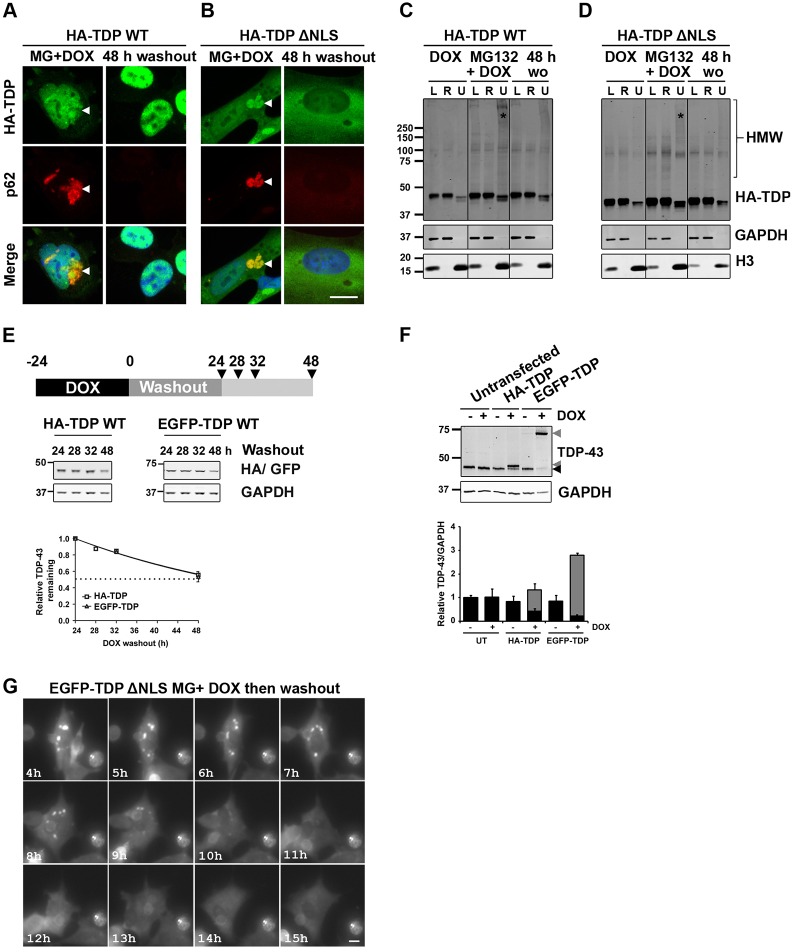
**TDP-43 macroaggregates are cleared upon the restoration of UPS function.** Immunocytochemical analysis of stable SH-SY5Y lines induced to form HA–TDP-43 WT (A) or HA–TDP-43 ΔNLS (B) aggregates (arrowheads) by a 48 h induction with DOX plus MG132 then fixed or subjected to MG132 washout (wo) after which the cells were left for a further 48 h. Scale bar: 10 µm. (C,D) Solubility fractionation of stable HA–TDP-43 WT (C) or HA–TDP-43 ΔNLS (D) SH-SY5Y lines either induced with DOX, induced to form aggregates by 48-h treatment with DOX plus MG132 or induced to form aggregates then subjected to MG132 washout after which the cells were left for a further 48 h. Lysate (L), RIPA-soluble (R) and urea-soluble (U) fractions are shown. Urea fractions are concentrated 10-fold. MG132 (*) increased the proportion of high-molecular-weight insoluble TDP-43 (HMW) and this was reversible upon MG132 washout (48 h). (E) Validation that EGFP-tagged TDP-43 WT is degraded over the same timecourse as HA–TDP-43 WT in stable SH-SY5Y cells (*t*_1/2_; HA–TDP-43 WT, 29.2 h; EGFP–TDP-43 WT, 29.3 h). (F) Validation that both HA- and EGFP-tagged TDP-43 WT (grey arrowheads) are functional and can regulate the level of endogenous TDP-43 (black arrowhead) in stable SH-SY5Y cells. Black bars represent endogenous TDP-43 and grey bars represent exogenous TDP-43. UT, untransfected. (G) Live-cell imaging of stable SH-SY5Y lines induced to form EGFP–TDP-43 ΔNLS aggregates by 48 h induction with DOX plus MG132 then subjected to washout and followed for a further 15 h. In cells that cleared aggregates, the aggregates fragmented before clearance. Scale bar: 10 µm.

We next sought to specifically investigate the handling of insoluble TDP-43. We used 1 µg/ml DOX plus MG132, which acts reversibly, to induce the formation of insoluble TDP-43 WT or ΔNLS cellular aggregates and then we followed the fate of those aggregates after washout of MG132. DOX was maintained in the medium at a concentration of 1 µg/ml during washout to ensure that our findings reflected TDP-43 aggregate handling in the face of continued expression of TDP-43. End-point imaging showed that for both WT (Fig. 6A) and ΔNLS TDP-43 (Fig. 6B), cells given a 48-h washout period after MG132 treatment were devoid of TDP-43-positive and p62-positive aggregates, in stark contrast to cells fixed directly after the MG132 treatment. The biochemical correlate of this was a reduction in detergent-resistant high-molecular-weight TDP-43 following MG132 washout (Fig. 6C,D). These findings might indicate that there is selective survival of cells without TDP-43 aggregates or that aggregated TDP-43 is either refolded or degraded following restoration of UPS function.

We therefore examined the clearance of aggregates in live cells, using stable SH-SY5Y cells transfected with EGFP-tagged TDP-43. We verified that the replacement of the N-terminal HA-tag with EGFP did not alter the timecourse of TDP-43 degradation by performing doxycycline pulse-chase experiments (Fig. 6E, *t*_1/2_; EGFP–�TDP WT, 29.3 h; HA�–TDP WT, 29.2 h). We also verified that EGFP-tagged TDP-43 was functional, as evidenced by its ability to downregulate endogenous TDP-43 (Ayala et al., 2011) (Fig. 6F). We next performed live-cell imaging of cells with MG132-induced aggregates of EGFP–TDP-43 ΔNLS, which indicated that most aggregate-laden cells died over a 15-h washout period (69.3%±5.6) and also that aggregates were rapidly cleared by the surviving cells (Fig. 6G; supplementary material Movie 1). Owing to the low fluorescence intensity of EGFP–TDP WT aggregates, their clearance could not readily be measured in living cells.

### TDP-43 aggregate clearance is due to the enhanced mobility of lower-order aggregated species, rather than the clearance of macroaggregated TDP-43

**Fig. 7.**
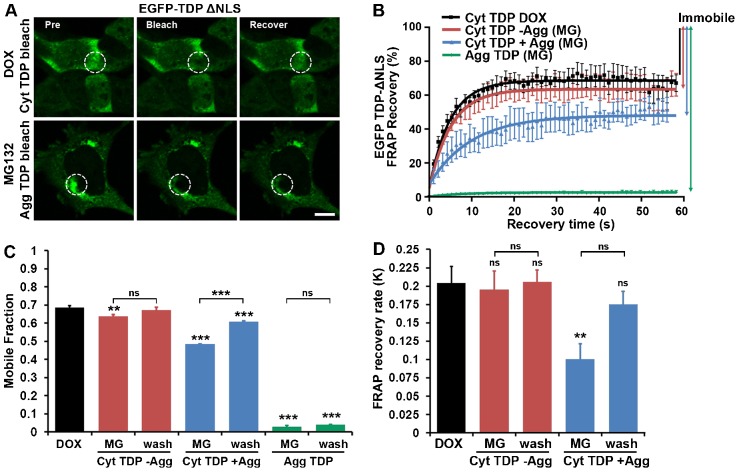
**TDP-43 aggregate clearance is due to enhanced mobility of lower-order aggregated species, rather than mobility of macroaggregated TDP-43.** (A) Confocal images acquired during FRAP. EGFP–TDP-43-ΔNLS showed rapid fluorescence recovery for diffuse cytosolic forms (Cyt TDP) but no recovery for aggregated forms (Agg TDP), indicating that macroaggregated TDP-43 is immobile and unlikely to readily exchange with the diffuse pool. The white dashed line encloses the bleached area. Scale bar: 10 µm. (B) FRAP recovery curves, with bleaching at *t* = 0 and 100% representing the pre-bleach fluorescence intensity. The fraction of cytosolic TDP-43 that was mobile was slightly decreased in cells that were treated with MG132 (MG) but were devoid of aggregates (Cyt TDP −Agg) and was greatly decreased in cells containing an aggregate (Cyt TDP +Agg). (C) The fraction of TDP-43 that was mobile under various conditions. The decrease in the mobile fraction in cells treated with MG132 and containing an aggregate (Cyt TDP +Agg) was rescued 8–10 h after washout (wash). There was no mobilisation of macroaggregated TDP-43 upon washout. (D) The rate of FRAP recovery [given as *K* = ln(2)/*t*_1/2_] for mobile species under various conditions. The FRAP rate was significantly decreased in cells that were treated with MG132 and contained an aggregate (Cyt TDP +Agg), which was reversed after washout. The bars represent means±s.e.m.; n.s., not significant. **P≤0.01, ***P≤0.001 between control and treated cells or as shown (one-way ANOVA, Bonferroni post-test).

Previous work on other aggregate-prone proteins indicates that some aggregates (e.g. juxta-nuclear quality control aggregates, JUNQ) are mobile, readily exchange with the diffuse protein pool and can be cleared by the UPS (Ben-Gedalya et al., 2011; Kaganovich et al., 2008). To determine whether this was true for TDP-43, we performed fluorescence recovery after photobleaching (FRAP) on diffuse and macroaggregated EGFP–TDP-43 ΔNLS (Fig. 7A) in stable SH-SY5Y cells. FRAP measures both the proportion of a given protein pool that is mobile and the rate of mobility by examining the migration of fluorescent proteins back into a region that has been bleached of fluorescence.

We examined cells that were induced to express EGFP–TDP-43 using 1 µg/ml DOX alone, cells induced to form aggregates with 1 µg/ml DOX plus MG132, and cells induced to form aggregates then washed out and left in fresh medium containing 1 µg/ml DOX without MG132 for 8–10 h. Firstly, by bleaching diffuse cytosolic EGFP–TDP-43 ΔNLS we found that the proportion of diffuse TDP-43 protein that was mobile was reduced by inhibition of the UPS, both in cells devoid of aggregates and, more strikingly, in cells that contained large aggregates (Fig. 7B,C; percentage of TDP-43 that was mobile: DOX only, 69%±0.9; MG132 without aggregates, 64%±1, *P*≤0.01; MG132 with aggregates, 48%±1.6, *P*≤0.001; ±s.e.m.). Furthermore, we found that the TDP-43 within the aggregates was almost completely immobile, with the bleached portions of the aggregates remaining dark for the duration of FRAP imaging and not visibly exchanging with the diffuse pool (Fig. 7A–C; percentage of TDP-43 that was mobile: 2.7%±0.1, *P*≤0.001). At 8–10 h after the washout of MG132, there was a significant increase in the proportion of diffuse TDP-43 that was mobile in cells still containing large aggregates, suggesting there was mobilisation or clearance of previously immobile species (percentage of TDP that was mobile: 48%±1.6 before and 61%±0.7 after washout, *P*≤0.001). However, direct bleaching of macroaggregated TDP-43 showed that this species was not mobilised, as it showed no increase in the mobile fraction after washout.

Next, we examined the rate of fluorescence recovery (the speed of molecular movement) for mobile species of TDP-43 protein (Fig. 7D). Diffuse TDP-43 in vehicle-treated cells showed rapid recovery (*t*_1/2_ = 3.4 s). Diffuse TDP-43 in cells exposed to UPS inhibition but without aggregates showed a similar rate of recovery (*t*_1/2_ = 3.6 s), whereas TDP-43 in cells with aggregates showed significantly slower recovery (*t*_1/2_ = 6.9 s). A decrease in the fluorescence recovery rate indicates increased binding or increased size of the TDP-43 particle, consistent with oligomerisation of TDP-43. At 8–10 h after the washout of MG132, there was an increase in the rate of recovery of diffuse TDP-43 in cells with large aggregates (*t*_1/2_ = 6.9 s before versus 4.0 s after washout), suggesting that there had been removal or dissociation of slow-moving species.

### TDP-43 aggregate clearance requires autophagy

**Fig. 8.**
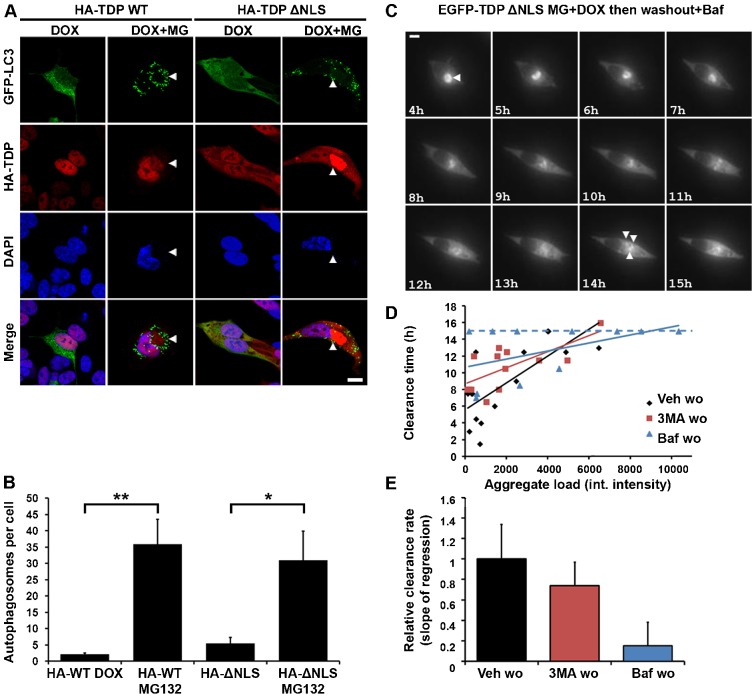
**TDP-43 aggregate clearance requires autophagy.** (A) Autophagosomes in cells containing TDP-43 aggregates. Stable SH-SY5Y lines were transfected with mCherry–GFP–LC3 and then induced to form HA–TDP-43 WT or ΔNLS aggregates (arrowheads) by a 48-h induction with DOX plus MG132. (B) Quantification of the number of GFP–LC3-positive autophagosomes per cell in cells treated as in A. The bars represent means±s.e.m. *P≤0.05, **P≤0.01 between control and treated cells (unpaired Student’s *t*-tests with Welch's correction). (C) Live-cell imaging of stable SH-SY5Y lines that were induced to form EGFP–TDP-43 ΔNLS aggregates (arrowheads) by a 48-h induction with DOX plus MG132, then subjected to washout and followed for a further 15 h in the presence of bafilomycin (Baf). Aggregates fragmented but were not cleared. (D) Regression analysis of the relationship between the aggregate load (integrated intensity of aggregate) and clearance time in the presence or absence of autophagy inhibitors during washout (wo) [3MA, 3-methyladenine; Baf, bafilomycin; Veh, vehicle; Pearson's *r* = 0.72 (Veh wo); 0.70 (3MA wo); 0.23 (Baf wo)]. Small aggregates were cleared rapidly in the absence, but not presence, of autophagy inhibitors. In Baf wo cells a distinct population of aggregates that were not cleared by the end of imaging (scored as 15 h) can be seen (dashed line). (E) Relative rates of clearance of EGFP–TDP-43 ΔNLS aggregates according to the slopes of the lines shown in D. Note that the slope for Baf wo includes the ‘non-clearing’ population. The results are not significant by one-way ANOVA. Scale bars: 10 µm.

Our findings suggested that during washout, slow-moving and immobile species were either resolved into mobile or faster-moving species, or were removed directly. Oligomeric or microaggregated species cannot be cleared directly by the UPS, and their clearance might require aggregate autophagy or ‘aggrephagy’ (Lamark and Johansen, 2012; Øverbye et al., 2007). Therefore, in order to test whether aggregate clearance involved autophagy, we first determined whether cells with TDP-43 aggregates contained autophagosomes, the double-membraned effectors of autophagy. Cells that were transfected with the autophagosome marker mCherry–GFP–LC3 showed a significant increase in the number of autophagosomes (GFP-positive puncta) in cells induced to form TDP-43 aggregates (1 µg/ml DOX plus MG132) compared to controls (Fig. 8A,B; autophagosomes per cell: WT, 36±8 MG132 versus 2±1 DOX, *P*≤0.01; ΔNLS, 31±9 MG132 versus 6±2 DOX, *P*≤0.05; ±s.e.m.).

We next examined the impact of autophagy inhibition on aggregate clearance, by performing live imaging. Cells were induced to form aggregates of EGFP–TDP-43 ΔNLS using 1 µg/ml of DOX plus MG132, and inhibitors of autophagy were added during the washout period (Fig. 8C). As discussed, the majority of cells with aggregates died over the course of imaging. Aggregate-containing cells that died had significantly greater aggregate load than those that lived (average aggregate integrated intensity was 7328±1661, versus 1961±522 in cells that lived, *P*≤0.01, Student’s *t*-test with Welch’s correction; ±s.e.m.). For cells that survived the period of imaging, we plotted the relationship between the load of aggregated protein and the rate of clearance from the cell (Fig. 8D). In the absence of autophagy inhibitors, we found a positive linear relationship between the aggregated protein load and the time taken for clearance (Fig. 8D, Veh). When autophagy inhibitors were present, macroaggregates were still disassembled into smaller discrete aggregates, suggesting an autophagy-independent disaggregation step (Fig. 8D, Baf; supplementary material Movie 2). However, these aggregates then persisted in the cells. The slope of the line relating initial aggregate load with clearance time therefore flattened in the presence of autophagy inhibitors (Fig. 8D,E, relative slope 3MA, 0.74; Baf, 0.15). The presence of aggregates that completely failed to be cleared over the 15-h timecourse in the presence of bafilomycin, regardless of their size (Fig. 8D, dashed line), showed that aggregate clearance was strongly dependent on autophagy.

## DISCUSSION

A number of genetic causes of ALS and FTD converge upon the pathways involved in TDP-43 protein degradation. Mutations in TDP-43 itself confer resistance to degradation (Kabashi et al., 2008; Rutherford et al., 2008; Sreedharan et al., 2008), whereas mutations in p62, ubiquilin-2 and VCP (Deng et al., 2011; Fecto et al., 2011; Watts et al., 2004) disrupt the autophagy and UPS pathways, both of which are involved in the degradation of TDP-43. However, the relative contribution of these pathways to the maintenance of TDP-43 proteostasis has been unclear. Previous studies provide conflicting evidence, finding either that wild-type TDP-43 is degraded predominantly by the UPS (Caccamo et al., 2009; Crippa et al., 2010; Wang et al., 2010; Zhang et al., 2010) or predominantly by autophagy (Urushitani et al., 2010), and none have specifically examined the degradation of aggregated TDP-43. Here, we demonstrate that the degradation of the soluble complement of full-length TDP-43 is mediated primarily by the UPS, whereas aggregated TDP-43 requires autophagic clearance.

As is the case for many proteins, cellular TDP-43 can exist as several different species with variable microscopical appearance and variable solubility and mobility profiles. In this study, we find evidence for soluble monomeric, soluble oligomeric, insoluble microaggregated and insoluble macroaggregated forms of TDP-43. Soluble monomer appears diffuse, is detergent-soluble and shows fast FRAP. Soluble oligomer also appears diffuse and is detergent-soluble but shows slow FRAP. Insoluble microaggregates also appear diffuse but are detergent-insoluble and immobile in FRAP experiments. Insoluble macroaggregates are evident only in UPS-inhibitor-treated cells, as immuno-detectable entities composed of detergent-insoluble high-molecular-weight species that show no FRAP mobility.

**Fig. 9.**
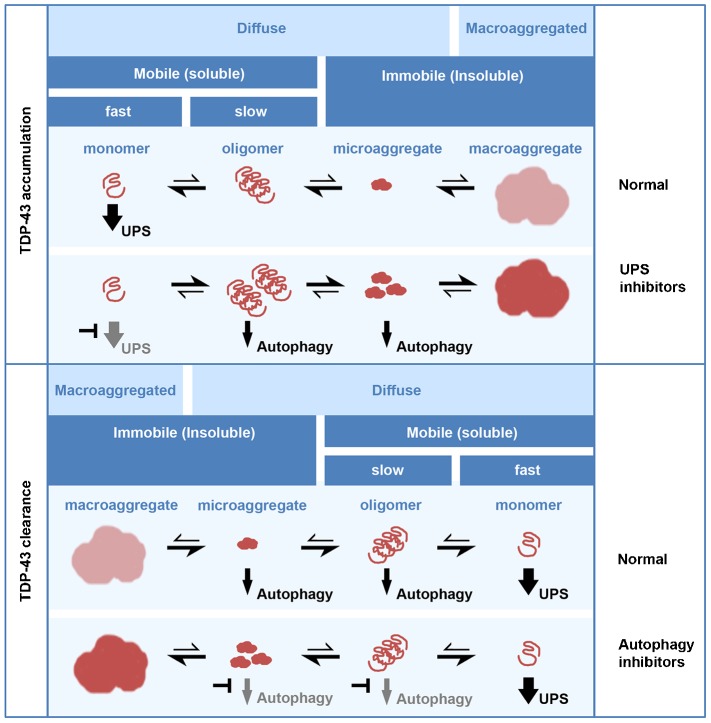
**A working model for the differential degradation of TDP-43 species.** Upper panel; under normal conditions, TDP-43 exists as several different species with variable appearance (diffuse or macroaggregated), solubility and mobility (fast or slow). When the UPS is functional, the removal of soluble monomer drives the equilibrium in favour of small soluble species and precludes the formation of macroaggregates. By contrast, UPS blockade drives the accumulation of oligomers and insoluble species, where insoluble species include ‘microaggregated’ and macroaggregated TDP-43; microaggregates appear visually diffuse but are immobile in FRAP experiments and macroaggregates are visible by immunofluorescence and immobile. Lower panel; a working model for TDP-43 clearance after macroaggregate formation. Under normal conditions macroaggregates are cleared by the induction of autophagy, which removes oligomeric and microaggregated species. Monomer is cleared by the UPS. Autophagy blockade prevents the removal of oligomer and microaggregates, although the fragmentation of macroaggregates still occurs.

Our proposed model for the existence of these species under different conditions is outlined in Fig. 9. Normally, in the presence of a functional UPS, the removal of soluble monomer serves to drive the equilibrium sufficiently to the left to favour small soluble species and to preclude the formation of macroaggregates. Therefore, in both cell culture models and tissue from human controls, only a small proportion of TDP-43 is detergent-insoluble basally (Neumann et al., 2006). By contrast, UPS blockade drives the accumulation of slow-moving oligomers and insoluble immobile TDP-43 species, which can appear diffuse (microaggregates) or clearly aggregated (macroaggregates). The clearance of macroaggregates involves fragmentation into smaller pieces, which is autophagy independent, and then the removal of lower-order species by autophagy. Oligomers, microaggregates or both can be substrates for autophagy, as autophagy blockade favours the continued existence of fragmented macroaggregates.

It has previously been shown that inhibition of the UPS can induce aggregates of full-length TDP-43 (Kim et al., 2012; van Eersel et al., 2011). However, to our knowledge, full-length TDP-43 has never been shown to aggregate in cells under conditions of autophagy inhibition alone. Similarly, ALS with TDP-43 proteinopathy can be modelled *in vivo* in mice by knockout of a proteasome subunit (Rpt3, also called PSMC4) but not by knockout of a key molecule in autophagy induction, Atg7 (Tashiro et al., 2012). This supports a model whereby the levels of soluble TDP-43 are predominantly regulated by the UPS and the accumulation of these species can lead to the ‘nucleation’ of aggregates. Thus, the impairment of proteasomal degradation in cell lines recapitulates the TDP-43 accumulation, aggregation and inclusion formation that occurs *in vivo*. The subsequent impairment of autophagy prevents the removal of aggregated TDP-43.

There has been fierce scientific debate amongst neurodegeneration researchers as to whether visible protein macroaggregates are the ‘toxic species’, are protective or are epiphenomena associated with, but not causative of, disease. The emerging consensus is that macroaggregates might be a ‘sink’ for the true toxic species, which are predominantly lower-order oligomers with greater mobility and surface area, and thus have enhanced potential for aberrant interactions (Caughey and Lansbury, 2003; Haass and Selkoe, 2007). Macroaggregates can, however, act as a surrogate to assess the levels of these oligomeric species. It is likely that the large TDP-43 macroaggregates that we report here are ‘aggresomes’, a conglomeration of smaller aggregates that are actively transported along microtubules to perinuclear regions (Kopito, 2000). Aggresome formation is dependent on several molecules whose genomic mutation is linked to ALS and FTD, including VCP, p62 and dynein–dynactin (Johnston et al., 2002; Puls et al., 2003; Seibenhener et al., 2004), as well as the ubiquilin-2-related molecule, ubiquilin-1 (Heir et al., 2006). Aggresomes are thought not only to sequester smaller aggregates to minimise their toxicity but also to coordinate aggregate removal by autophagy, a process known as ‘aggrephagy’ (Lamark and Johansen, 2012). Aggrephagy is therefore emerging as a key target for drug discovery in ALS and FTD (Thomas et al., 2013).

In this study, we show for the first time that full-length TDP-43 macroaggregates are labelled by both K48- and K63-linked polyubiquitin chains. Ubiquitin chains with these different linkages are thought to direct proteins towards different fates owing to the specificity of binding partners for specific linkages. K48-linked polyubiquitin chains are associated with UPS degradation (Xu et al., 2009). By contrast, K63-linked chains are associated with an autophagic fate. The autophagic adaptor protein p62 is selective for K63-linked chains (Seibenhener et al., 2004) and acts to recruit LC3-positive autophagosomes (Pankiv et al., 2007). Ubiquilin-2 can bind proteins labelled with either type of ubiquitin chain linkage (Rothenberg et al., 2010). The presence of both types of chain on TDP-43 aggregates could be consistent with a model in which K48–ubiquitin-labelled substrates cannot be degraded by the UPS and are subsequently labelled with K63–ubiquitin for aggrephagy (Yamamoto and Simonsen, 2011).

An autophagic mode of clearance of aggregated TDP-43 echoes that of other neurodegenerative disease proteins. Soluble fragments of wild-type huntingtin are degraded proteasomally but aggregated expanded-repeat fragments are not accessible to the proteasome and are degraded by autophagy (Qin et al., 2003). Mutant androgen receptor, the protein that causes spinal and bulbar muscular atrophy (SBMA), is toxic to flies and this toxicity is enhanced when autophagy is blocked together with the UPS (Pandey et al., 2007).

Although autophagy is inducible in response to a heavy burden of aggregated proteins (Ding et al., 2007; Iwata et al., 2005; Laussmann et al., 2011; Rideout et al., 2004), in our model autophagy induction was unable to clear TDP-43 macroaggregates in the face of continued UPS inhibition. Dual treatment with inhibitors of the UPS and autophagy revealed that autophagy had been serving to reduce the aggregated TDP-43 burden caused by UPS inhibition alone. However, complete aggregate clearance only occurred after washout of the UPS inhibitor, indicating that autophagy could not fully compensate for UPS impairment. Although we used low-dose chronic UPS inhibition to attempt to more closely mimic the slow degenerative process in humans, the degree of UPS impairment is likely to far exceed that which could be caused by aging or by the genetic mutations detected in some ALS and FTD cases. Therefore, the activation of autophagy remains a promising therapeutic approach to treating ALS and FTD in humans. Indeed, autophagy activation has been shown to clear α-synuclein inclusions in primary postmitotic neurons even when there is continued moderate inhibition of the UPS using lactacystin (Rideout et al., 2004). Excitingly, in a mouse model of FTD based on TDP-43 overexpression, rapamycin [an inhibitor of mTOR, which therefore activates (de-represses) autophagy] was used to induce the clearance of TDP-43, increasing motor neuron survival and motor function (Wang et al., 2012).

As we age, the efficiency of both the UPS and autophagy decline (Gamerdinger et al., 2009; Tydlacka et al., 2008; Zhou et al., 2003). However, in aged individuals without ALS or FTD, TDP-43 aggregates do not commonly accumulate. In people affected by ALS and FTD, TDP-43 inclusions form and remain at the end of life, leading us to speculate that, in addition to normal aging, there is secondary impairment of the UPS or autophagy, promoting the accumulation of misfolded species and preventing their removal. Augmenting UPS function could help to restore TDP-43 proteostasis and, where UPS impairment persists, the activation of autophagy might reduce the aggregated TDP-43 protein burden. Therefore, identifying molecules that selectively enhance each of these processes, and that can be used in combination to reduce TDP-43 accumulation, is a valid therapeutic strategy.

## MATERIALS AND METHODS

### Plasmids

Constructs are shown schematically in Fig. 1A. These constructs allow doxycycline-inducible expression of tagged (HA or EGFP) TDP-43 wild type (WT), nuclear localisation sequence-deleted [ΔNLS (Δ82–98)] or C-terminal fragment [CTF (181–414)]. All constructs were generated by Gateway cloning (Invitrogen Life Technologies, Carlsbad, CA). AttB-flanked PCR products were subcloned into pDONR221 using BP clonase II, then into pDEST30 using LR clonase II, according to the manufacturer’s instructions (Invitrogen Life Technologies). N-terminal HA.11-tagged TDP-43 WT, ΔNLS and CTF were amplified from existing constructs (Nishimura et al., 2010). N-terminal EGFP-tagged TDP-43 WT was first generated using pEGFP-C1 (*Xho*I/*Bam*HI) then subcloned into Gateway. EGFP-tagged TDP-43 ΔNLS was generated by site-directed mutagenesis of the WT construct in pDONR (QuikChange II, Stratagene). Primer sequences are available on request.

All constructs were verified in-house by DNA sequencing using Big-Dye^®^ Terminator v1.1 (Invitrogen Life Technologies, Paisley, UK) on an ABI3130 genetic analyser (Applied Biosystems Pty Ltd, Warrington, UK). Sequence chromatograms were analysed using Geneious v5.4.6 (Biomatters, Auckland, NZ). All primers were obtained from Sigma-Aldrich (Dorset, UK). mCherry–GFP–LC3 plasmid (Pankiv et al., 2007) was kindly supplied by Terje Johansen (University of Tromsø, Norway). FLAG–Ub plasmid (Tan et al., 2008) was a kind gift of Lim Kah-Leong (National University of Singapore).

### UPS and autophagy modifying drugs

3MA (used at 10 mM), bafilomycin (400 nM) and lithium chloride (10 mM) were purchased from Acros Organics (Geel, Belgium). MG132 (0.5 µM), epoxomicin (100 nM) and rapamycin (0.2 µg/ml) were purchased from Cayman Chemicals (Cambridge, UK). Trehalose (100 mM) was purchased from Sigma-Aldrich.

### Mammalian cell line culture, transfection, plating and induction

Stable tetracycline-inducible (Tet-ON) clonal cell lines were generated in human neuroblastoma SH-SY5Y cells (CRL-2266; ATCC, Middlesex, UK) and human embryonic kidney HEK293 (T-REx HEK293, #R710-07, Invitrogen Life Technologies), using the T-REx system. Briefly, stable clonal lines transfected with pcDNA6/TR (encoding a constitutively expressed Tet-repressor protein) were transfected with pDEST30-TDP-43 constructs using Lipofectamine 2000, selected using 600 µg/ml geneticin and clonally isolated.

SH-SY5Y cells were maintained in DMEM/F12 plus Glutamax, supplemented with 10% fetal bovine serum (FBS) (Tet-free, S0115T, BioChrom AG, Berlin, Germany), 100 U/ml penicillin and 100 µg/ml streptomycin. HEK293 cells (stable Tet-ON) were maintained in DMEM plus Glutamax, supplemented with 10% FBS (Tet-free), 100 U/ml penicillin and 100 µg/ml streptomycin. All cells were maintained at 37°C, under 5% CO_2_.

Transient transfection of mCherry–GFP–LC3 into stable SH-SY5Y lines was performed using Lipofectamine 2000, as per the manufacturer’s instructions. Transient transfection of FLAG–Ub into stable HEK293 lines was performed using Fugene HD (Promega, Southampton, UK) as per the manufacturer’s instructions. For experimental analyses, SH-SY5Y lines were plated at 75,000 cells/cm^2^, HEK293 lines were plated at 25,000 cells/cm^2^ and TDP-43 expression was induced using 1 µg/ml DOX, unless otherwise stated. All reagents were purchased from Invitrogen Life Technologies unless otherwise stated.

### Immunofluorescence and imaging

For immunofluorescence analyses, SH-SY5Y lines were plated onto untreated 13-mm coverslips (1.5 thickness). HEK293 lines were plated onto poly-D-lysine-treated coverslips. After treatment, cells were fixed in 4% paraformaldehyde (PFA, VWR International Ltd, Leicestershire, UK) for 10 min and rinsed with phosphate-buffered saline (PBS). The cells were incubated with primary antibody overnight at 4°C, then with fluorescent secondary antibodies for 6 h at room temperature, each diluted in PBS with 0.2% Triton X-100 and 1% serum (goat or donkey as appropriate). For thioflavin T staining, cells were stained for 10 min with freshly prepared and filtered 0.1% thioflavin T (Acros Organics) in PBS and rinsed. After immunodetection, the coverslips were mounted onto slides using fluorescence mounting medium (Dako, Carpenteria, CA) and left to harden overnight.

The antibodies used for immunofluorescence were against TDP-43 (mouse: sc-100871, 1∶500, Santa Cruz Biotechnology, Heidelberg, Germany; rabbit: 1078-2-2-AP, 1∶500, ProteinTech Europe, Manchester, UK), HA.11 (mouse; MMS-101P, 1∶1000, Covance, Leeds, UK: rabbit; #3724, 1∶1000, Cell Signaling Technology, Beverly, MA), p62 (#610833, 1∶1000, BD Biosciences, San Jose, CA), ubiquitin (Z0458, 1∶200, Dako UK Ltd, Ely, UK), K48-linked ubiquitin (#05-1307, 1∶500, Millipore, Temecula, CA), K63-linked ubiquitin (#05-1308, 1∶500, Millipore), ubiquilin-2 (sc-100612, 1∶2000, Santa Cruz Biotechnology; also detects ubiquilin-1 by western bloting). Invitrogen Life Technologies goat secondary antibodies (Alexa-Fluor-488−anti-mouse-IgG, A11001; Alexa-Fluor-568−anti-rabbit-IgG, A11011) or Jackson ImmunoResearch donkey secondary antibody (Alexa-Fluor-649−anti-mouse-IgG, 715-495-150) were used at 1∶500.

Wide-field imaging was performed using a Zeiss Axiovert S100 (Carl Zeiss Ltd, Hertfordshire, UK) with a ×63/NA 1.25 Plan Neofluar oil immersion objective, fitted with a CoolSnap EZ digital camera (Photometrics, Tucson, AZ). Confocal images were acquired using a Leica TCS SP5 confocal laser scanning microscope (Leica Microsystems, Buckingham, UK) with a ×63/NA 1.4 Plan Apo oil immersion objective and a pinhole of 95.6 µm (Airy 1). Images (1024×1024 pixels) were acquired at 400 Hz with a line averaging of 8, using Leica LAS-AF software and 405 nm, 488 nm and 633 nm laser lines.

Line intensity profiles were quantified using the colour profiler plug-in (Dimiter Prodanov) in ImageJ (version 1.45e, NIH, Bethesda, USA, http://rsb.info.nih.gov/ij/) on merged RGB images for a representative experiment. GFP–LC3 autophagosomes were quantified using the Granularity tool in Metamorph (v. 7.7, Molecular Devices, Wokingham, UK) and the granules were normalised to the number of transfected cells by manual counting of GFP-positive cells. 113–622 granules were counted in 20–50 transfected cells in a representative experiment.

### Doxycycline pulse–chase assay

The degradation rates of the various TDP-43 constructs were investigated by doxycycline (DOX) pulse–chase as described previously (Ravikumar et al., 2002). Briefly, TDP-43 expression was induced for 24 h using 10 ng/ml DOX to achieve almost maximal expression (Fig. 1D,E). For washout, the cells were rinsed twice in fresh DOX-free medium. For the initial characterisation of the degradation rate in HEK293 cells, the samples were harvested just before DOX washout (0 h) and 24, 48 and 72 h after washout (Fig. 2A). For the validation of degradation rates in SH-SY5Y cells, samples were harvested 24, 28, 32 and 48 h after washout (Fig. 2B). To investigate the degradation pathways for TDP-43 in HEK293 cells, samples were harvested just before DOX washout (0 h) then inhibitors, activators or vehicle controls were added after 24 h of washout and samples were taken after a further 48 h (Fig. 3A). The cells were harvested into 1×SDS loading buffer [Laemmli buffer (Laemmli, 1970) without Bromophenol Blue or reducing agents] and were boiled for 10 min before freezing at −20°C. Wells treated with MG132, epoxomicin or bafilomycin were plated at twice the density used for degradation assays, to reduce toxicity (Bar et al., 2004). Degradation timecourse data were fitted to a one-phase exponential decay and plotted using GraphPad Prism 6.02 (GraphPad Software, San Diego, CA).

### Quantitative PCR

Total RNA was extracted and DNase-treated using the RNeasy Lipid Tissue Mini Kit (Qiagen, West Sussex, UK) and cDNA was reverse transcribed using oligo(dT) primers with SuperScript^®^ III First-Strand Synthesis SuperMix (Invitrogen Life Technologies). Quantitative PCR was performed using FASTStart SYBR Green Mastermix on a 7900HT Fast Real-Time PCR System (Applied Biosystems) running SDS v.2.3 software. The primers that were used to detect HA–TDP WT and ΔNLS transcripts were 5′-TACCCATACGATGTTCCAGATTAC-3′ and 5′-GCATGCAGAATTCCTTCTACC-3′. HA–TDP CTF transcripts were quantified using the same HA-specific forward primer with the reverse primer 5′-CCCGTACTGAGAGAAGAACT-3′. *GAPDH* acted as a reference transcript, using the primers 5′-CAGCCTCAAGATCATCAGCA-3′ and 5′-GGCATGGACTGTGGTCATGAG-3′. PCR cycling conditions were: activation, 10 min at 95°C; cycling, 15 s at 95°C, 30 s at 60°C and 30 s at 72°C for 40 cycles; followed by the thermal denaturation protocol. The expression levels of HA–TDP transcripts relative to *GAPDH* transcripts (reference), with normalisation to no DOX (control), were determined using the Pfaffl method (Pfaffl, 2001).

### Solubility fractionation

The sequential biochemical fractionation of cellular proteins was performed as described previously for ALS and FTD brain homogenates (Sampathu et al., 2006), with several modifications for cells. Pellets were initially resuspended in low-salt (LS) buffer, and were sequentially resuspended in the following buffers (volumes are shown relative to the LS volume): Triton X-100, 200%; sarkosyl, 30%; urea, 40%. Centrifugation was performed at 14,000 rpm for 30 min at 4°C. The myelin flotation buffer step was omitted.

RIPA/urea solubility fractionation was performed as described by Winton and colleagues (Winton et al., 2008), using centrifugation at 14,000 rpm for 30 min at 4°C and omitting sonication. The final (urea) pellet was resuspended in 10% of the original lysis volume.

### Western blotting and densitometry analysis

Protein concentrations were quantified using the BioRad DC Protein Assay (BioRad, Hemel Hempstead, UK) and equivalent protein was loaded for each sample (HEK293, 5 µg; SH-SY5Y, 10 µg). For solubility experiments, whole-lysate protein was quantified and the equivalent liquid volume of the soluble and insoluble fractions was loaded. Gels were transferred onto nitrocellulose using the iBlot (Invitrogen Life Technologies), stained with Ponceau S and then blocked in TBS with 0.05% Tween-20 (TBS-T) and 5% non-fat dried milk (NFDM, Sigma-Aldrich) for 30 min. The blots were probed overnight at 4°C with primary antibody, then for 3 h at room temperature with secondary antibodies (all in TBS-T plus 1% NFDM). The blots were scanned on the Li-Cor Odyssey gel scanner (Li-Cor Biotechnology, Cambridge, UK) and were then reprobed (without stripping) for loading-control proteins using the same antibody conditions and scanning protocol.

Antibodies used for blotting were against TDP-43 (mouse: sc-100871, 1∶1000), HA.11 (#3724, 1∶1000), Histone H3 (H0164, 1∶10,000, Sigma-Aldrich), p62 (#610833, 1∶1000), GAPDH (G9545, 1∶1000, Sigma-Aldrich), GFP (mouse: sc-9996, 1∶1000, Santa Cruz), LC3B (#2775, 1∶500, Cell Signaling Technology) and Dylight fluorescent secondary antibodies (35521, goat anti-mouse-IgG–Dylight-680, 1∶5000; 35568, goat anti-rabbit-IgG–Dylight-700, 1∶10,000, Fisher Scientific UK Ltd, Leicestershire, UK).

The blot images in TIF format were quantified using the gel analyser tool in ImageJ. Integrated band intensities were normalised to the band intensities of loading controls and also to relative input for the solubility assays.

### TDP-43 aggregate clearance assays

The clearance of TDP-43 aggregates was assessed by RIPA/urea solubility fractionation and immunofluorescence (HA-tagged constructs) and live-cell imaging and fluorescence recovery after photobleaching (FRAP) (EGFP-tagged constructs). For all aggregate clearance assays, stable SH-SY5Y TDP-43 WT or ΔNLS cells were plated at 100,000 cells/cm^2^ and were left to recover overnight. TDP-43 expression was induced with DOX for 24 h and then aggregate formation was induced using DOX with 0.5 µM MG132 for a further 48 h. For washout experiments, the cells were then washed and left in fresh medium containing DOX without MG132, in the presence or absence of inhibitors for the stated time periods.

### Live-cell imaging

Live imaging was used to assess whether EGFP–TDP-43 ΔNLS aggregates were cleared. Stable SH-SY5Y cells were plated onto Hi-Q4 dishes (Ibidi GmbH, Germany). Immediately before imaging, the cells were washed and left in fresh medium without MG132, containing DOX and either vehicle (0.05% DMSO), 3MA (10 mM) or bafilomycin (400 nM).

Epifluorescent and phase images (1280×960 pixels) of selected cells with aggregates were acquired twice per hour for 15 h using a BioStation IM-Q (Nikon UK Ltd, Surrey, UK) fitted with a ×20/NA 0.5 Plan Fluor objective and maintained at 37°C, under 5% CO_2_. The image sets were analysed using ImageJ. The aggregate load was defined as the total integrated density (area×intensity) of all aggregates in a given cell, with aggregate boundaries determined by intensity thresholding. The clearance time was defined as the time at which aggregates were indistinguishable from diffuse TDP-43.

### Fluorescence recovery after photobleaching

Stable SH-SY5Y EGFP–TDP-43 WT or ΔNLS cells were plated onto 18-mm coverslips (1.5 thickness). To investigate protein mobility after MG132 washout, the cells were washed and left in fresh medium containing DOX, but without MG132, for 8–10 h before FRAP.

FRAP was performed using a Nikon A1 plus laser scanning confocal microscope fitted with an environmental chamber maintained at 37°C (Solent Scientific, Segensworth, UK). Confocal images were acquired using a ×60/NA 1.4 Apo oil immersion objective, a confocal pinhole of 34.8 µm (Airy 1.2) and a 488-nm laser line. FRAP was performed using NIS-Elements AR software (v. 4.00.04). Images of 512×512 pixels were acquired. Five pre-bleach frames were acquired with a laser power of 1–3%, before bleaching EGFP–TDP-43 (70 msec, 50% argon laser) using a square region of interest (ROI) of 2 µm^2^. Fluorescence recovery was followed for 1 min, with a laser power of 1–3% to minimise acquisition bleaching.

Data analysis was performed using ImageJ with the Bio-Formats plugin (Linkert et al., 2010). The mean intensity at each timepoint was measured for the bleached ROI as well as for ROIs in the background and in a neighbouring unbleached cell to correct for acquisition bleaching. FRAP recovery curves for the bleached ROI were calculated by first subtracting the background at each timepoint and correcting for acquisition bleaching, then normalising to pre-bleach and bleach using the equation
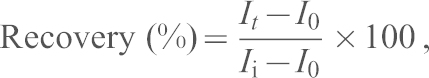
where *I_t_* is the fluorescence intensity of the ROI at a given time, *I*_0_ is the fluorescence intensity of the ROI immediately after bleaching and *I*_i_ is the average fluorescence intensity of the ROI before bleaching. The data were fitted to a one-phase exponential decay and plotted using GraphPad Prism 6.02.

### Statistical analysis

Data handling and graphical representations were performed using Microsoft Excel unless otherwise stated. Statistical analyses as described in the figure legends were performed using GraphPad Prism 6.02. One-way ANOVA was performed after Bartlett's testing for equal variance. For both one- and two-way ANOVA, Bonferroni post-tests were used to compare the selected data sets. Two-tailed Student’s *t*-tests were performed after *F*-testing for equal variance and Welch's correction was applied where stated. The Pearson correlation test was performed after D'Agostino and Pearson normality testing. Statistical significance was set at *P*≤0.05 (*P≤0.05; **P≤0.01; ***P≤0.001). All figures were prepared using Adobe Photoshop CS3.

## Supplementary Material

Supplementary Material
