# Supplementary material for: Differential roles of the ubiquitin proteasome system and autophagy in the clearance of soluble and aggregated TDP-43 species
Source: J Cell Sci. 2014 Mar 15;127(6):1263–78. doi: 10.1242/jcs.140087 (PMC3953816; doi:10.1242/jcs.140087)
Supplement: Supplementary Material [file supp_127_6_1263__index.html]

Differential roles of the ubiquitin proteasome system and autophagy in the clearance of soluble and aggregated TDP-43 species — Supplementary Material 

# Differential roles of the ubiquitin proteasome system and autophagy in the clearance of soluble and aggregated TDP-43 species

## JCS140087 Supplementary Material

**Files in this Data Supplement:**

- **Supplementary Material**
