# Supplementary material for: Differential roles of the ubiquitin proteasome system and autophagy in the clearance of soluble and aggregated TDP-43 species
Source: J Cell Sci. 2014 Mar 15;127(6):1263–78. doi: 10.1242/jcs.140087 (PMC3953816; doi:10.1242/jcs.140087)
Supplement: Supplementary Material [file supp_127.6.1263_JCS140087.pdf]

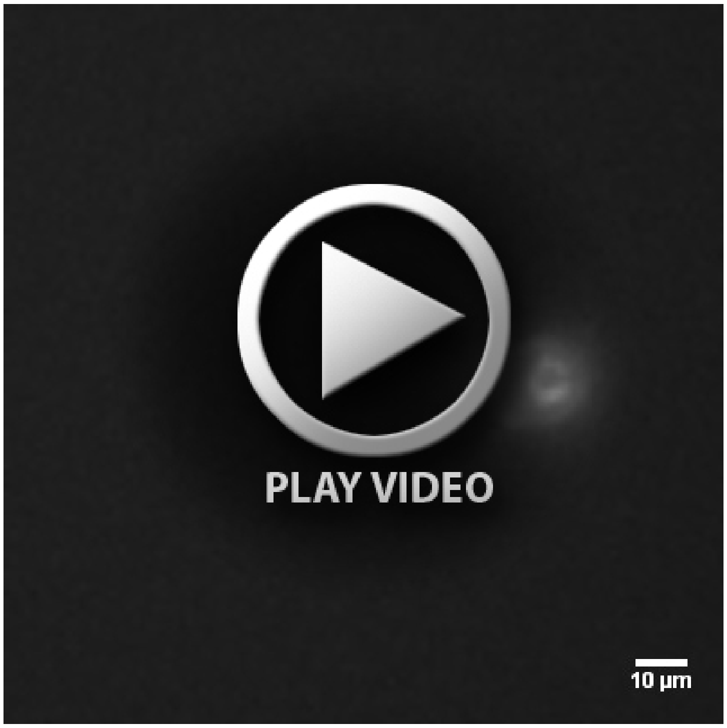

Movie S1. TDP-43 macroaggregate clearance. Live cell imaging of stable SH-SY5Y lines induced to form EGFP-TDP-43 ΔNLS aggregates by 48-h induction with DOX plus MG132 then washed out and followed for a further 15 in the presence of vehicle. Large aggregates fragmented and were cleared. Each frame of the video represents 1 h of imaging, total 15 h.

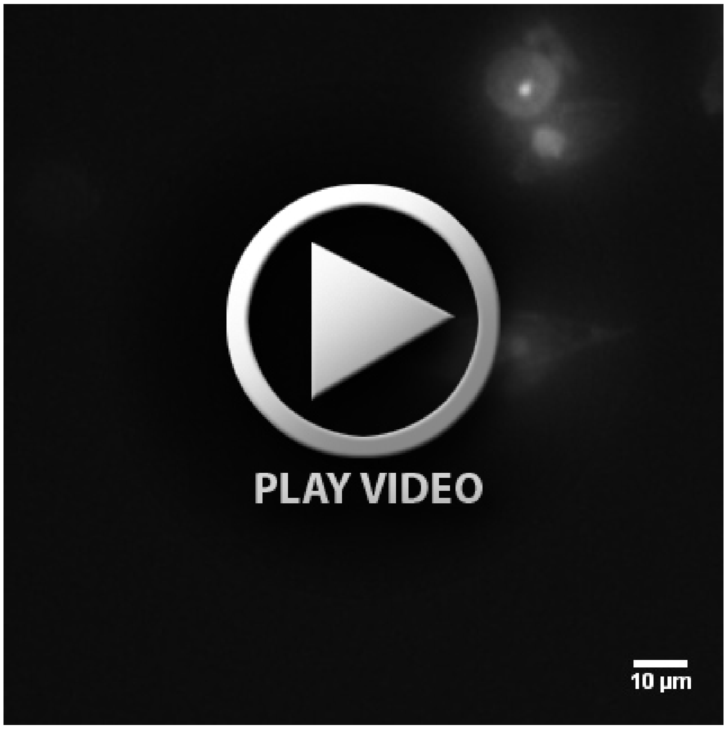

Movie S2. TDP-43 macroaggregate clearance requires autophagy. Live cell imaging of stable SH-SY5Y lines induced to form EGFP-TDP-43 ΔNLS aggregates by 48 h induction with DOX plus MG132 then washed out and followed for a further 15 in the presence of bafilomycin. Large aggregates fragmented but fragments persisted under autophagy inhibition. Each frame of the video represents 1 h of imaging, total 15 h.
